# Fine-Needle Aspiration Cytology of Epithelioid Leiomyoblastoma

**DOI:** 10.1080/13577149977802

**Published:** 1999-03

**Authors:** Karen Sue Thompson, Joanne Jensen, Cesar V. Reyes

**Affiliations:** 1 Veterans Affairs Hospital Hines, and Loyola University Stritch School of Medicine Maywood Illinois USA; 2 578-113 Room D121 P.O. Box 5000 Fifth & Roosevelt Hines IL 60141 USA

## Abstract

*Purpose.*  Epithelioid leiomyoblastomas comprise the majority of gastric sarcomas and are uncommon in other parts of the gastrointestinal tract. Diagnosis of this lesion by fine-needle aspiration cytology has been occasionally described in the literature. Two additional cases are herein reported.

*Subjects .*  A 66-year old male with an omental mass and a 47-year old male with a perihepatic tumor.

*Results and Discussion.* Cytologic materials in both cases showed predominantly round or epithelioid cells, along with polygonal to spindle cells, occuring singly and in clusters, with oval to spindle-shaped nuclei.The nuclei were monotonous, usually banal, and centrally-located with only focal suggestion of pleomorphism and rare mitosis. Eosinophilic cytoplasm was noted in most of the cells, some demonstrating vacuolation. Electron microscopy supported a primitive smooth cell derivation of the neoplastic cells.

*Conclusions.*  The cytomorphology of the tumors of the two cases reported here is not adequately known. More cases need to be collected and studied.


*Sarcoma* (1999) **3**, 5–9

**ORIGINAL ARTICLE**

### Fine-needle aspiration cytology of epithelioid leiomyoblastoma

KAREN SUE THOMPSON, JOANNE JENSEN & CESAR V. REYES

*Veterans Affairs Hospital, Hines, and Loyola University Stritch School of Medicine, Maywood, Illinois, USA*

**Abstract**

*Purpose.* Epithelioid leiomyoblastomas comprise the majority of gastric sarcomas and are uncommon in other parts of the gastrointestinal tract. Diagnosis of this lesion by fine-needle aspiration cytology has been occasionally described in the literature. Two additional cases are herein reported.

*Subjects.* A 66-year old male with an omental mass and a 47-year old male with a perihepatic tumor.

*Results and Discussion.* Cytologic materials in both cases showed predominantly round or epithelioid cells, along with polygonal to spindle cells, occuring singly and in clusters, with oval to spindle-shaped nuclei. The nuclei were monotonous, usually banal, and centrally-located with only focal suggestion of pleomorphism and rare mitosis. Eosinophilic cytoplasm was noted in most of the cells, some demonstrating vacuolation. Electron microscopy supported a primitive smooth cell derivation of the neoplastic cells.

*Conclusions.* The cytomorphology of the tumors of the two cases reported here is not adequately known. More cases need to be collected and studied.

**Key words:** *leiomyoblastoma, epithelioid, cytology, fine-needle aspiration cytology, sarcoma.*

### Introduction

Epithelioid leiomyoblastoma refers to a round cell differentiation in smooth muscle tumors that may present as a diagnostic dilemma and misinterpretation for carcinoma. The clinical behavior is difficult to predict although the microscopic findings of one or more mitoses per high-power field indicate a potentially malignant tumor. The majority of the lesions are benign, and only one quarter are malignant. About 95% of cases originate in the stomach. The lesion is frequently seen in the mid-to-late adult life and is more prevalent in men than women. Its cytomorphology has been characterized but only in a few published cases in the literature.[1–9] The following paper is a report of two additional cases diagnosed by fine needle aspiration biopsy (FNAB) with ultrastructural evaluations.

### Subjects

### *Case 1*

A 49-year-old white man with a history of acute hemorrhagic pancreatitis secondary to trauma presented to Hines VA Hospital for further evaluation of a recurrent pseudocyst. Past medical history included an adrenocortical carcinoma removed at age 29 years, familial polyposis and total colectomy at age 39 years. Additionally a Puestow procedure was performed one year earlier for the hemorrhagic pancreatitis during which time an intra-abdominal sarcoma was incidentally discovered and resected.

An ultrasound showed an intra-abdominal mass around the liver (Fig. 1).

The fine needle aspiration biopsy evaluated with Papanicolaou smears and hematoxylin-eosin stained cell block preparation demonstrated fairly monotonous, non-epithelial tumor cells with a moderate amount of pale almost clear cytoplasm, and rare mitotic figures, consistent with epithelioid cell stromal tumor, probably malignant (Fig. 2). Comparison between this material and those of the intra-abdominal 'sarcoma' resected one year earlier indicated identical tumors. Ultrastructural analysis of the FNAB rinsing sediment demonstrated ill-formed intercellular junctions, few intracytoplasmic microfilaments, and rare pinocytotic vesicles, supporting a primitive smooth muscle origin of the tumor cells.

Resection of a 4×2.5×2-cm tumor tissue from the round ligament and hepatic capsule was well tolerated. The gross specimen exhibited variegated, somewhat nodular cut surfaces. Light and electron microscopy of the tumor tissue supported the diagnosis of epithelioid leiomyoblastoma.

The patient did very well on follow-up. However,

Correspondence to: Dr C. V. Reyes, 578-113, Room D121, P.O. Box 5000, Fifth & Roosevelt, Hines, IL 60141, USA. Tel. (708) 343-7200 x1295; Fax (708) 216-2588.

1357-714X/99/010005-05 $9.00 © 1999 Taylor & Francis Ltd

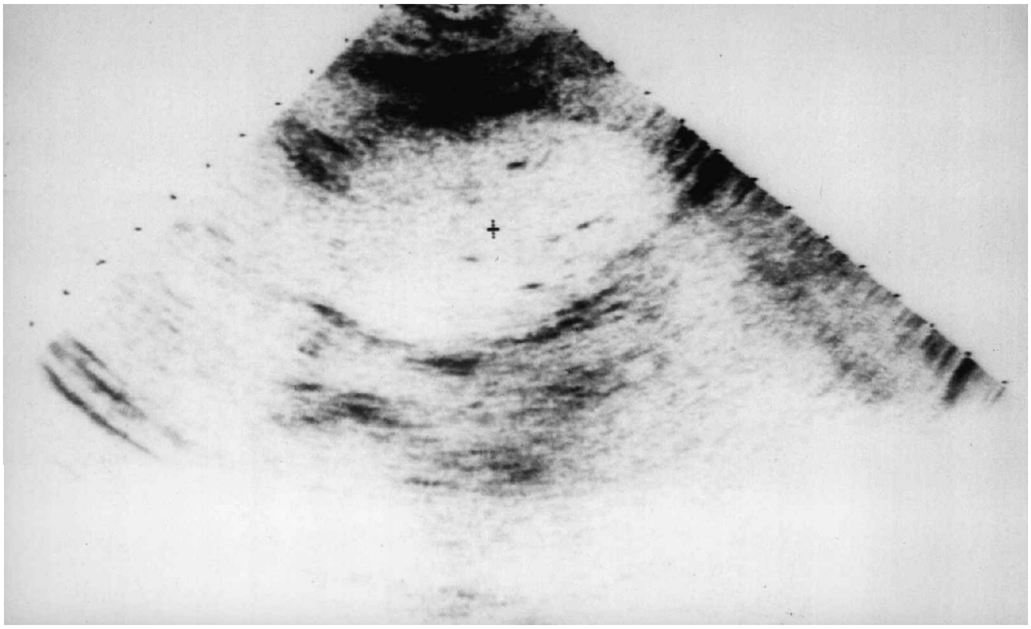

**Fig. 1.** *Ultrasound imaging reveals a mass lesion around the liver (+).*

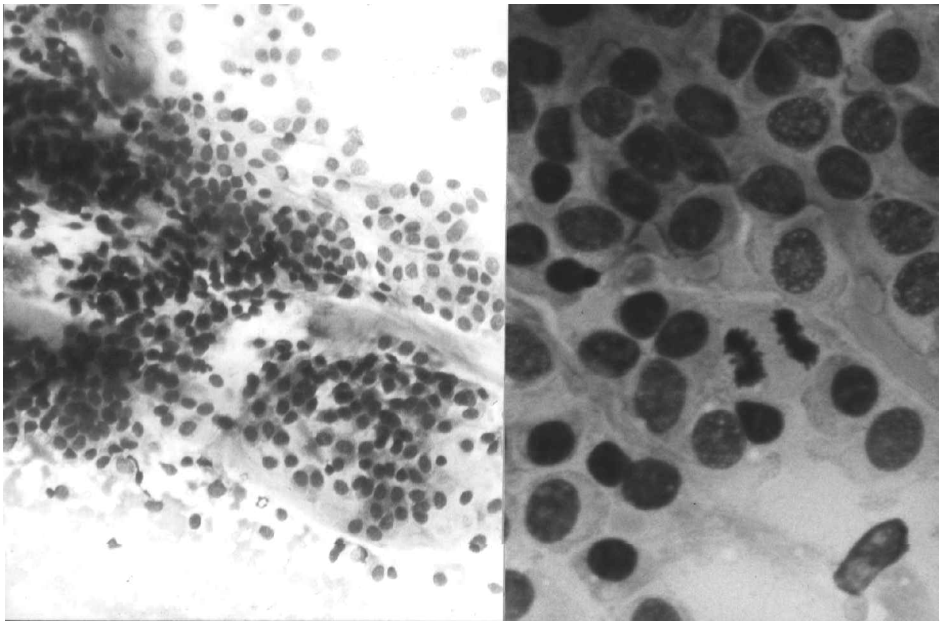

**Fig. 2.** *The neoplastic cells are epithelioid and invariably non-cohesive with focal nuclear pleomorphism and occasional mitosis (Diff Quik stain, ×100 and ×400).*

he continued to experience several clinical problems, including recurrent pancreatic pseudocysts, diagnosis and resection of somastatinoma arising in an ectopic pancreas in the duodenum, and ampulla of Vater adenocarcinoma treated with Whipple's procedure.

The intra-abdominal leiomyoblastoma has not recurred in the last 16 years.

*Case 2*

A 75-year-old white man presented with a left upper quadrant abdominal pain associated with fullness and tenderness just above the umbilicus. Past medical history included hypertension, congestive heart failure secondary to coronary artery disease, adult-onset diabetes mellitus, hypothyroidism, post-infarct dementia, and resected peripancreatic sarcoma nine years earlier.

A computer tomographic scan displayed a large mass in the right upper quadrant, attached to the greater omentum and anterior abdominal wall (Fig. 3). FNAB was performed and the Diff Quik preparation showed a cellular smear of fairly monotonous, dyscohesive, epithelioid cells with some eosinophilic, almost acellular fibrohyaline stroma, and some nuclear pleomorphism. Papanicolaou stained smears emphasized the non-epithelial nature of the tumor cells, minimal pale cytoplasm, and absence of mitosis (Fig. 4). Hematoxylin eosin-stained cell block exhibited myxoid

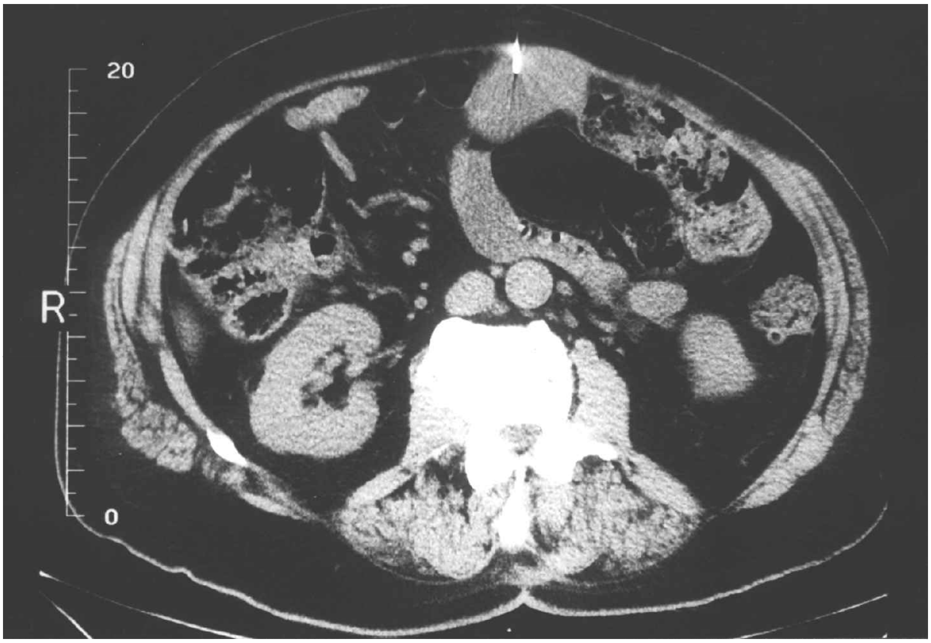

**Fig. 3.** *Computer tomographic scan-guided FNAB of an omental fat/abdominal wall mass shows the fine needle in place.*

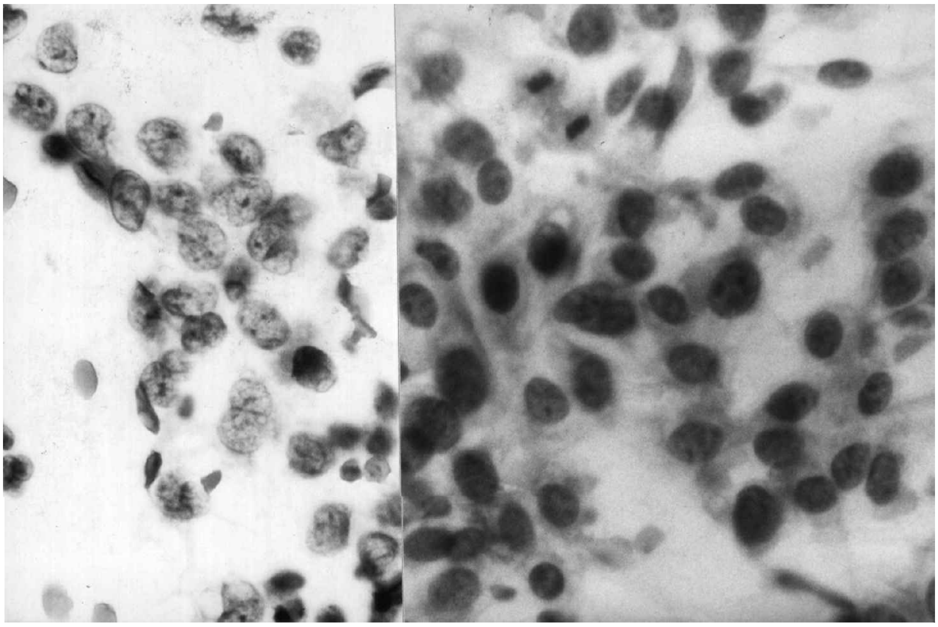

**Fig. 4.** *Cell block preparation highlights uniform nonepithelial cells with minimal pale cytoplasm and mitosis (Papanicolaou stain, ×400).*

stroma, mild cellular pleomorphism, and abnormal mitosis. Keratin-negative and desmin-positive cells were demonstrated. Electron microscopy showed similar findings as observed in Case 1 and supported a primitive smooth muscle origin of the neoplastic cells (Fig. 5). Subsequent resection of 5×4.8×4.5-cm greater omental tumor was uncomplicated. Light and electron microscopy and immunostaining studies of the resected tumor reaffirmed the FNAB interpretation. Correlation of the FNAB material with the resected peripancreatic sarcoma nine years earlier also provided identical microscopic findings.

The patient is free of recurrence after two years.

### Discussion

The term 'leiomyoblastoma' was first coined by Stout in 1962 for a group of bizarre, round-cell smooth muscle tumors and was applied to avoid strict connotation of benignancy or malignancy. Its use has recently waned in some quarters, however, because of the contention that these tumors should be approached much like the conventional smooth muscle neoplasm. Other names applied to the lesion in the literature are 'gastrointestinal stromal tumor' and 'epithelioid smooth muscle tumor'.

Epithelioid leiomyoblastomas are found most commonly in the gastric wall and occasionally in the

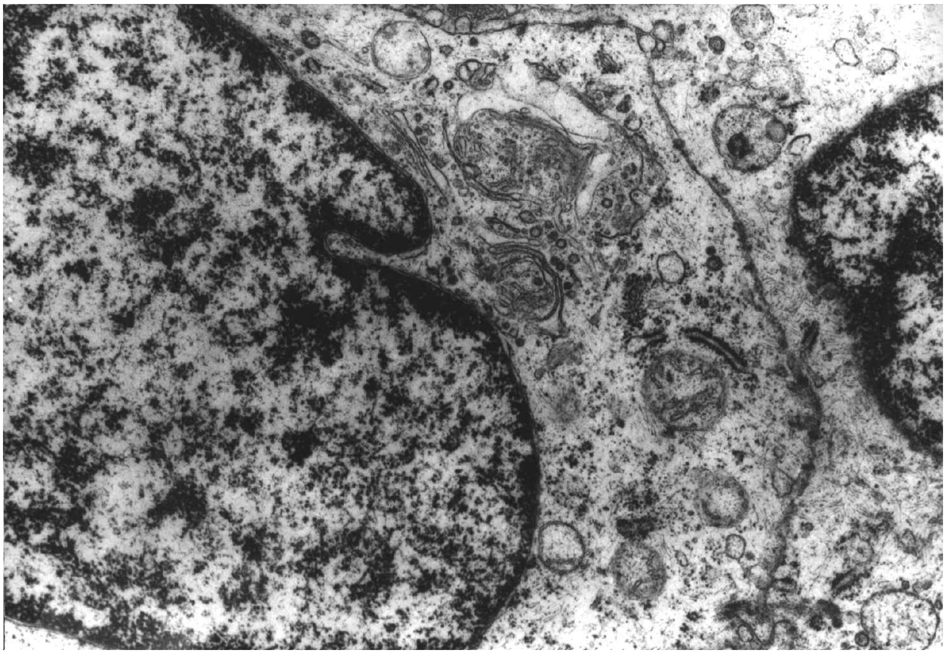

**Fig. 5.** *Rare ill-formed junction structures, few intracytoplasmic microfilaments, and occasional pinocytotic vesicles are seen ultrastructually and support a primitive smooth muscle origin (×13 500).*

esophagus, intestine, mesentery, omentum, retroperitoneum, mediastinum, uterus, vulva, and skin. They usually manifest in mid-to-late adult life and are rare before 20 years of age. Males are more frequently affected than females. The majority are present as upper gastrointestinal bleeding, abdominal pain or mass.

The histogenesis is controversial. The tumors rarely demonstrate a clear-cut muscle differentiation; however, smooth muscle features have been well documented. A neural origin has also been suggested because of variable S100 positivity.[8]

Only a few published reports of FNAB evaluation of these tumors exist. The notable cytologic characteristics include singly scattered, round to polygonal cells; round to oval, centrally placed hyperchromatic nuclei; eosinophilic granular cytoplasm; and frequent vacuolated cytoplasm[5] sometimes with signet-ring cell appearance.[4] These vacuoles are probably degenerative and do not contain lipid, mucin, or immunoglobulin. Oncocytic change has also been described.[6] On rare occasions the stroma can be myxoid.[7] Nuclear atypia, pleomorphism, and nucleoli are variable.

A biphasic pattern of rounded, epithelioid cells and short, spindle smooth muscle cells, in varying proportions, with transitional forms, aids in the correct diagnosis of smooth muscle origin. Malignancy is suggested by increased dyscohesion, pleomorphism, coarse chromatin, prominent nucleoli, scant cytoplasm, and necrosis. The finding of one or more mitoses per high-power field strengthens this impression regarding malignancy.[1,2]

The subject of malignancy in epithelioid leiomyoblastoma has been widely debated and has been influenced by the following factors, namely: the duration of clinical symptoms, location and size of the tumor, and the number of mitosis per high power field. Unfavorable prognosis is usually defined when the symptoms are of more than six months duration, the tumor size is greater than 6 cm, when it is extragastric in location, and when there are five or more mitoses per 50 high power fields.[2,9]

Ultrastructurally, the findings include thin myofilaments, cytoplasmic and subplasmalemmal dense bodies, pinocytotic vesicles, basal lamina and intercellular junction structures. However, these features are less frequent in epithelioid leiomyoblastoma than in smooth muscle tumors. The clear or vacuolated cytoplasm is virtually indiscernible with electron microscopy and it is now considered as an artifact of fixation.[8,9]

In summary, immunohistochemical and electron microscopic findings are varied and researchers continue to debate the histogenesis of epithelioid leiomyoblastoma. The entity of ‘epithelioid smooth muscle tumors’ is accepted by many and is classified further into benign and malignant forms. The criteria for malignancy depend on a combination of factors and mitotic count alone is not sufficient. To date, very few epithelioid leiomyoblastomas diagnosed by FNAB have been reported in the literature. Herein, we presented two additional cases. The cytomorphology of these tumors is still not adequately known, and more cases need to be collected and studied.

### References

1 Smith DN, Silverman JF, Raab SS, *et al.* Fine-needle aspiration cytology of hepatic leiomyosarcoma. *Diagn Cytopathol* 1991; 7:321–7.

2 Tao L-C, Davidson DD. Aspiration biopsy cytology of smooth muscle tumors. A cytologic approach to the differentiation between leiomyosarcoma and leiomyoma. *Acta Cytol* 1993; 37:300–8.

3 Insabato L, D'Armiento FP. Diagnostic value of intranuclear vacuoles in gastric leiomyoblastoma. *Diagn Cytopathol* 1992; 8:548–9.

4 Kung ITM, Yuen RWS, Hwang ST. Fine-needle aspiration of gastric leiomyoblastoma metastatic to the subcutis. *Diagn Cytopathol* 1991; 7:68–71.

5 Nguyen G-K. Cytopathologic aspects of leiomyoblastoma in fine-needle aspiration biopsy. Report of two cases. *Acta Cytol* 1983; 27:173–7.

6 Nance K, Reddick RL. Epithelioid leiomyosarcoma of the small intestine with oncocytic change. *Arch Pathol Lab Med* 1987; 111:1181–2.

7 Lagrange W. Fine needle aspiration biopsy of myxoid variant of malignant leiomyoblastoma metastatic to the liver. *Acta Cytol* 1988; 32:443–6.

8 Blei E, Gonzalez-Crussi F. The intriguing nature of gastric tumors in Carney's triad: Ultrastructure and immunhistochemical observations. *Cancer* 1992; 69:292–300.

9 DeMay RM. *The Arts & Science of Cytopathology*. Chicago: ASCP Press, 1996, Vol. 2, p. 578.
